# *IGLL5* is correlated with tumor‐infiltrating immune cells in clear cell renal cell carcinoma

**DOI:** 10.1002/2211-5463.13085

**Published:** 2021-02-05

**Authors:** Zhi‐Nan Xia, Xing‐Yuan Wang, Li‐Cheng Cai, Wen‐Gang Jian, Cheng Zhang

**Affiliations:** ^1^ Department of Urology The First Affiliated Hospital of Harbin Medical University China

**Keywords:** CIBERSORT, clear cell renal cell carcinoma, ESTIMATE, IGLL5, tumor microenvironment, tumor‐infiltrating immune cells

## Abstract

Renal cell carcinomas (RCCs) account for about 90% of renal tumors, and their major histological subtype is ccRCC (clear cell RCC). Increasing evidence has indicated that the tumor microenvironment plays a significant role in the occurrence and development of ccRCC. In this study, we used ESTIMATE and CIBERSORT computational methods to calculate the proportion of immune and stromal components and the rate of TICs (tumor‐infiltrating immune cells) in 539 ccRCC samples from The Cancer Genome Atlas database. By examining the intersection of the differentially expressed genes obtained by the protein–protein interaction network and Cox regression analysis, we identified only one overlapping gene: *IGLL5* (immunoglobulin lambda‐like polypeptide 5). We report that *IGLL5* expression is correlated with TICs. Furthermore, our immunoinfiltration analyses revealed that three types of TIC are positively correlated with *IGLL5* expression. *IGLL5* may have potential as a prognostic biomarker of ccRCC.

## Graphical abstract

We used computational methods to calculate the proportion of immune and stromal components and the rate of tumor‐infiltrating immune cells in clear cell renal cell carcinoma samples from The Cancer Genome Atlas database. One gene, *IGLL5*, was filtered between the PPI network and prognostic genes. Immunoinfiltration analyses revealed that three types of tumor‐infiltrating immune cell were positively correlated with *IGLL5* expression. In summary, *IGLL5* may have potential as a novel prognostic biomarker of clear cell renal cell carcinoma.

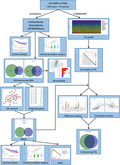

**Abbreviations**
ccRCC: clear cell renal cell carcinoma
DEG: differentially expressed gene
DR: down‐regulated
FC: fold change
FDR: false discovery rate
GO: Gene Ontology
GSEA: gene set enrichment analysis
ICI: immune checkpoint inhibitor
IGLL5: immunoglobulin lambda‐like polypeptide 5
KEGG: Kyoto Encyclopedia of Genes and Genomes
OS: overall survival
PPI: protein–protein interaction
RCC: renal cell carcinoma
TCGA: The Cancer Genome Atlas
TIC: tumor‐infiltrating immune cell
TME: tumor microenvironment
UR: up‐regulated

Renal cell carcinomas (RCCs) are malignant tumors that originated from renal tubular epithelial cells. RCCs account for about 90% of renal tumors, and their major histological subtype is clear cell RCC (ccRCC) [1]. In recent decades, the incidence rate of RCCs has increased in the majority of countries. It is estimated that 400 000 new cases of RCCs are diagnosed annually worldwide, resulting in more than 175 000 deaths. The incidence rate and mortality rate rank third in urological oncology universally [2, 3]. Radical nephrectomy is the first choice for early localized renal cancer, although it is still unable to tackle the problem of tumor recurrence or distant metastasis in more than 20% of patients postoperatively [4]. Meanwhile, RCCs have the characteristics of tolerance to radiotherapy and chemotherapy. As a consequence, it is highly essential to explore factors related to the prognosis and therapy of RCCs.

Tumor microenvironment (TME) has markedly attracted the attention of the tumor research community and has been recognized as a key factor in tumor progression in existing studies [5, 6, 7]. TME is a complex system of many groups of cells, including recruited immune and inflammatory cells and resident stromal cells, such as fibroblasts, neuroendocrine cells, adipose cells, among others [8]. A previous study showed that tumor‐infiltrating immune cells (TICs) in TME are a promising indicator of prognosis and therapeutic effects. Highly immune‐infiltrated TME is associated with adverse outcomes of ccRCC (e.g., higher tumor stage, higher recurrence rate, and mortality) [9]. Scholars have demonstrated that compositions of TICs are correlated with effects on immunotherapeutic interventions [10], confirming the effect of TICs on immunotherapy.

To date, The Cancer Genome Atlas (TCGA) database and bioinformatics have provided novel strategies for performing research on TME. An algorithm called “Estimation of Stromal and Immune cells in Malignant Tumours using Expression data (ESTIMATE)” was proposed to use gene expression data to calculate the proportion of stromal and immune cells in tumor samples [11], and this algorithm has been widely used to predict the proportion of immune and stromal cells in TMEs in different types of cancer [12, 13, 14]. In research conducted by Luo *et al*. [15], the ESTIMATE algorithm was first applied to ccRCC. Recently, more studies have concentrated on the application of ESTIMATE in ccRCC [16, 17, 18]. However, filtration conditions of hub genes were wide in previous studies, and they scarcely assessed the correlation between genes and TICs. In the present research, we obtained the differentially expressed genes (DEGs) by the ESTIMATE and then achieved hub genes from these DEGs by construction of a protein–protein interaction (PPI) network. Afterward, hub genes were combined with prognosis‐related genes to obtain the prognosis‐related hub genes, and finally, only one gene, immunoglobulin lambda‐like polypeptide 5 (*IGLL5*), complied with the requirements. In addition, we used CIBERSORT, a computational method that can estimate the relative proportion of each immune composition in a tumor sample, to calculate the ratios of TICs [19] (Fig. 1).

**Fig. 1:**
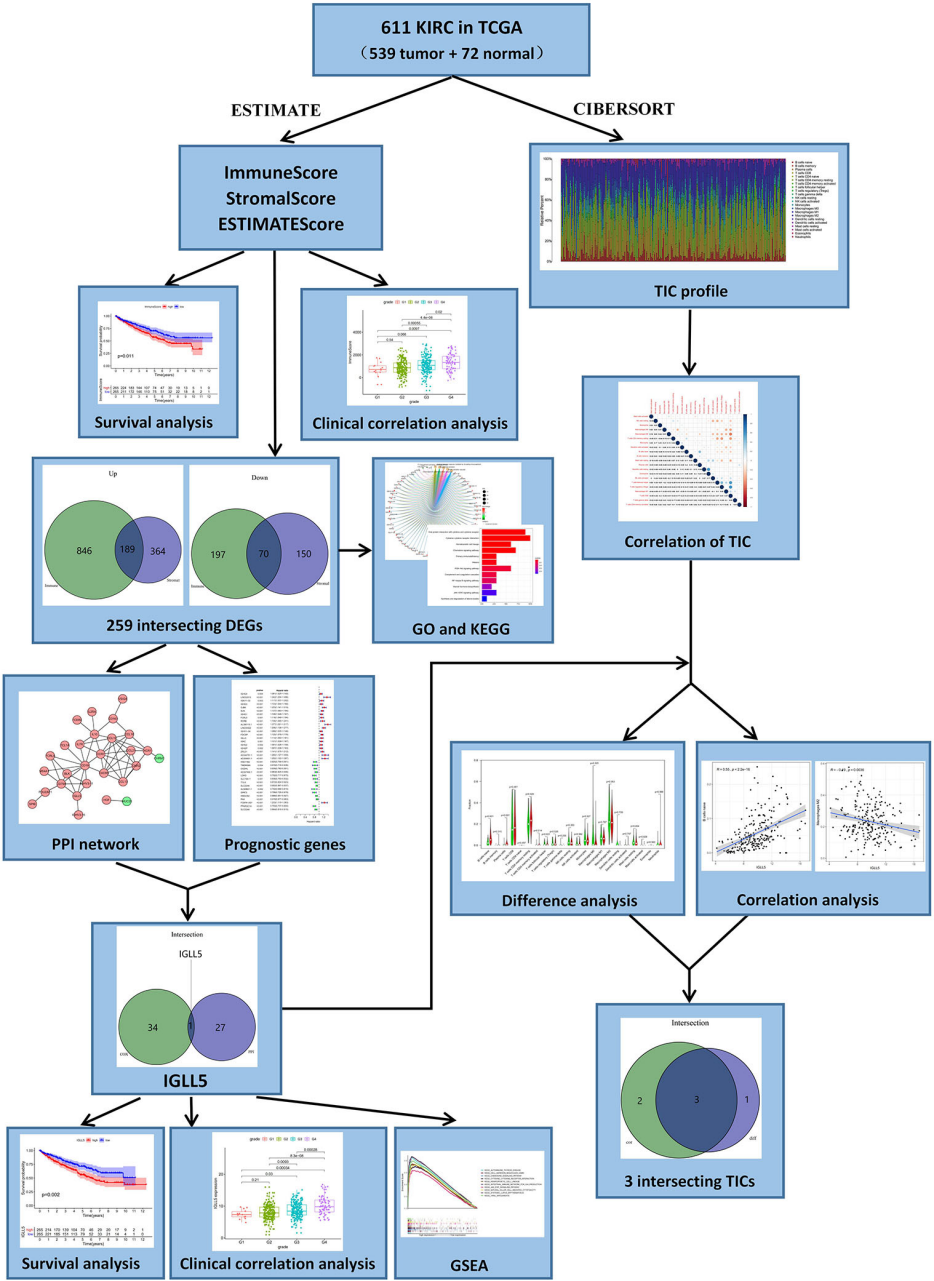
The flowchart of this experiment.

## Results

### Scores and the survival of patients with ccRCC are correlated

**Fig. 2:**
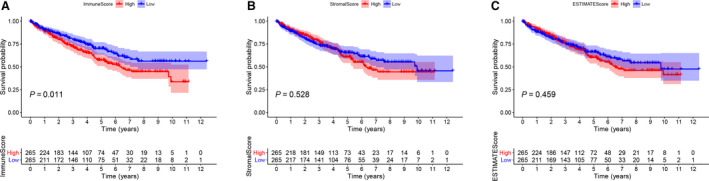
Correlation between scores and the survival of patients with ccRCC. (A) Kaplan–Meier survival analysis for ImmuneScore (*P* = 0.011). The red line represents the HS group, and the blue line represents the LS group. (B) Kaplan–Meier survival analysis for StromalScore (*P* = 0.528). (C) Kaplan–Meier survival analysis for ESTIMATEScore (*P* = 0.459).

We extracted the transcriptome and clinical data of 611 cases from the TCGA Kidney Renal Clear Cell Carcinoma database, with participation of 539 ccRCC and 72 normal samples. To evaluate the immune scores, stromal scores and ESTIMATE scores for each sample, we used the ESTIMATE. With higher ImmuneScore or StromalScore, TME involved more immune or stromal components, and the summation of ImmuneScore and StromalScore was presented by the ESTIMATEScore, which represented the comprehensive scale of these two components in TME. On the basis of the median score, the tumor samples were divided into high score (HS) and low score (LS) groups. Then we conducted the survival analysis to evaluate the correlation between scores and survival. The Kaplan–Meier plot indicated that ImmuneScore was correlated with overall survival (OS), and poorer prognosis of cases in the HS group was noted (Fig. 2A). StromalScore and ESTIMATEScore indicated that OS was longer in the LS group compared with that in the HS group; however, no significant differences were noted between the two groups (Fig. 2B,C). These results suggested that ImmuneScore could better reflect the prognosis of cases with ccRCC.

### Correlation between scores and the clinicopathological characteristics of patients with ccRCC

**Fig. 3:**
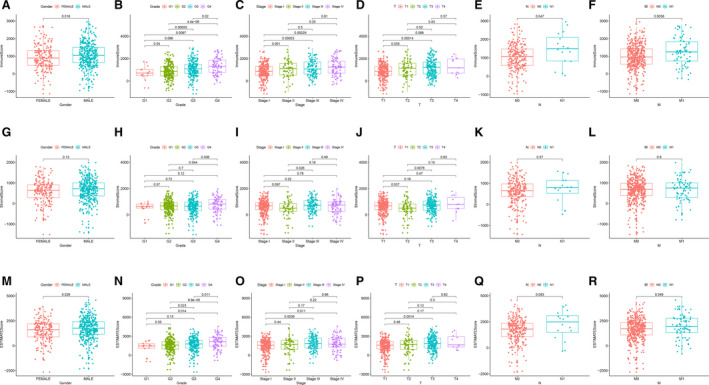
Correlation between scores and clinicopathological characteristics of patients with ccRCC. (A–F) ImmuneScore and sex (*P* < 0.05), Fuhrman grade (G1–G4, G2–G3, G2–G4, G3–G4; *P* < 0.05), clinical stage (stages I–IV; *P* < 0.05), T stage (T1–T3; *P* < 0.05) and N and M stages (*P* < 0.05). (G–L) StromalScore and sex, Fuhrman grade (G2–G4, G3–G4; *P* < 0.05) and TNM stage. (M–R) ESTIMATEScore and sex (*P* < 0.05), Fuhrman grade (G1–G4, G2–G3, G2–G4, G3–G4; *P* < 0.05), clinical stage (stages I–IV; *P* < 0.05), T stage (T1–T3; *P* < 0.05), N stage (*P* = 0.083) and M stage (*P* < 0.05).

To determine dependency of scores on clinicopathological characteristics, we attempted to investigate the clinical data of individuals with kidney renal clear cell carcinoma retrieved from TCGA. The Wilcoxon rank‐sum test was used for testing the correlation. As shown in Fig. 3, male patients with ccRCC had higher ImmuneScore (*P* = 0.016) and ESTIMATEScore (*P* = 0.029) than female patients with ccRCC (Fig. 3A,M), whereas the difference in StromalScore between male and female patients with ccRCC was not statistically significant (Fig. 3G). ImmuneScore and ESTIMATEScore increased with a higher rank order of Fuhrman grades (G1–G4, G2–G3, G2–G4, G3–G4; *P* < 0.05; Fig. 3B,N) and clinical stages (stages I–IV, *P* < 0.05; Fig. 3C,O). In addition, ImmuneScore and ESTIMATEScore increased with an increase in tumor size, except for T4 (T1–T3, *P* < 0.05; Fig. 3D,3P), and the probable reason may be that the sample size in T4 is not large enough, which reduces the statistical power to detect a significant difference. Moreover, patients with ccRCC with lymph node metastases and distant metastases had higher ImmuneScore (N and M stages, *P* < 0.05) and ESTIMATEScore (M stage, *P* < 0.05; N stage, *P* = 0.083) than patients without metastases (Fig. 3E,F,Q,R). Increased StromalScore was associated with an elevation in Fuhrman grade (G2–G4, G3–G4; *P* < 0.05; Fig. 3H). Nevertheless, StromalScore was associated with no significant difference in none of the other clinical parameters (Fig. 3I–L). Hence the proportion of immune and stromal components could be associated with the invasion and metastasis of patients with ccRCC.

### Gene Ontology and KEGG pathway enrichment analyses of DEGs

**Fig. 4:**
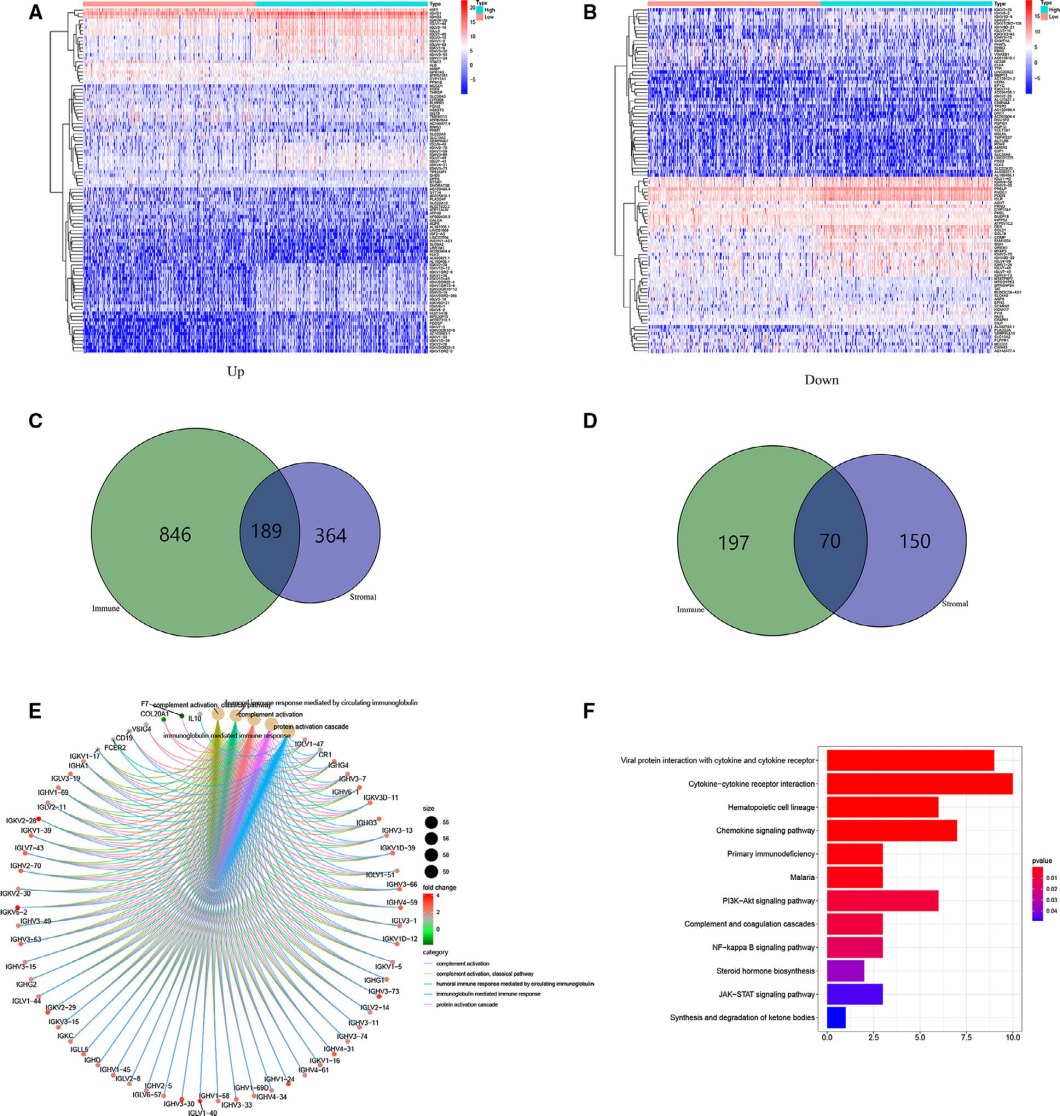
Analysis of DEGs. (A, B) Heatmaps for the most significant 100 DEGs based on logFC (50 UR and DR genes in each sample) in ImmuneScore (A) and StromalScore (B) (|logFC| > 1, FDR < 0.05). (C, D) Venn diagrams showing concurrently UR and DR DEGs in ImmuneScore (C) and StromalScore (D). (E, F) GO and KEGG enrichment analyses of DEGs (*P* < 0.05).

With respect to classification of patients mentioned earlier, we attempted to conduct the differential analysis by Wilcoxon test, and we identified 1491 DEGs for ImmuneScore, involving 1035 up‐regulated (UR) and 267 down‐regulated (DR) genes. Meanwhile, we identified 773 DEGs by the same process for StromalScore, including 553 UR and 220 DR genes (|logFC| > 1, false discovery rate [FDR] < 0.05; Fig. 4A,B). A Venn diagram was used to explore genes associated with TME, and we noted 259 intersecting genes in ccRCC samples, involving 189 UR genes and 70 DR genes in the HS group compared with LS groups (Fig. 4C,D).

To determine the roles of the DEGs, we conducted Gene Ontology (GO) and Kyoto Encyclopedia of Genes and Genomes (KEGG) functional enrichment analyses. The results of GO functional analyses demonstrated the enrichment of DEGs mainly in complement activation, immune response mediated by immunoglobulin and protein activation pathways (Fig. 4E). Furthermore, the pathways involved in tumor immune response and ccRCC tumorigenesis were identified in the KEGG pathway enrichment analyses (Fig. 4F).

### Establishment of the PPI network of DEGs and detection of the prognostic hub genes

**Fig. 5:**
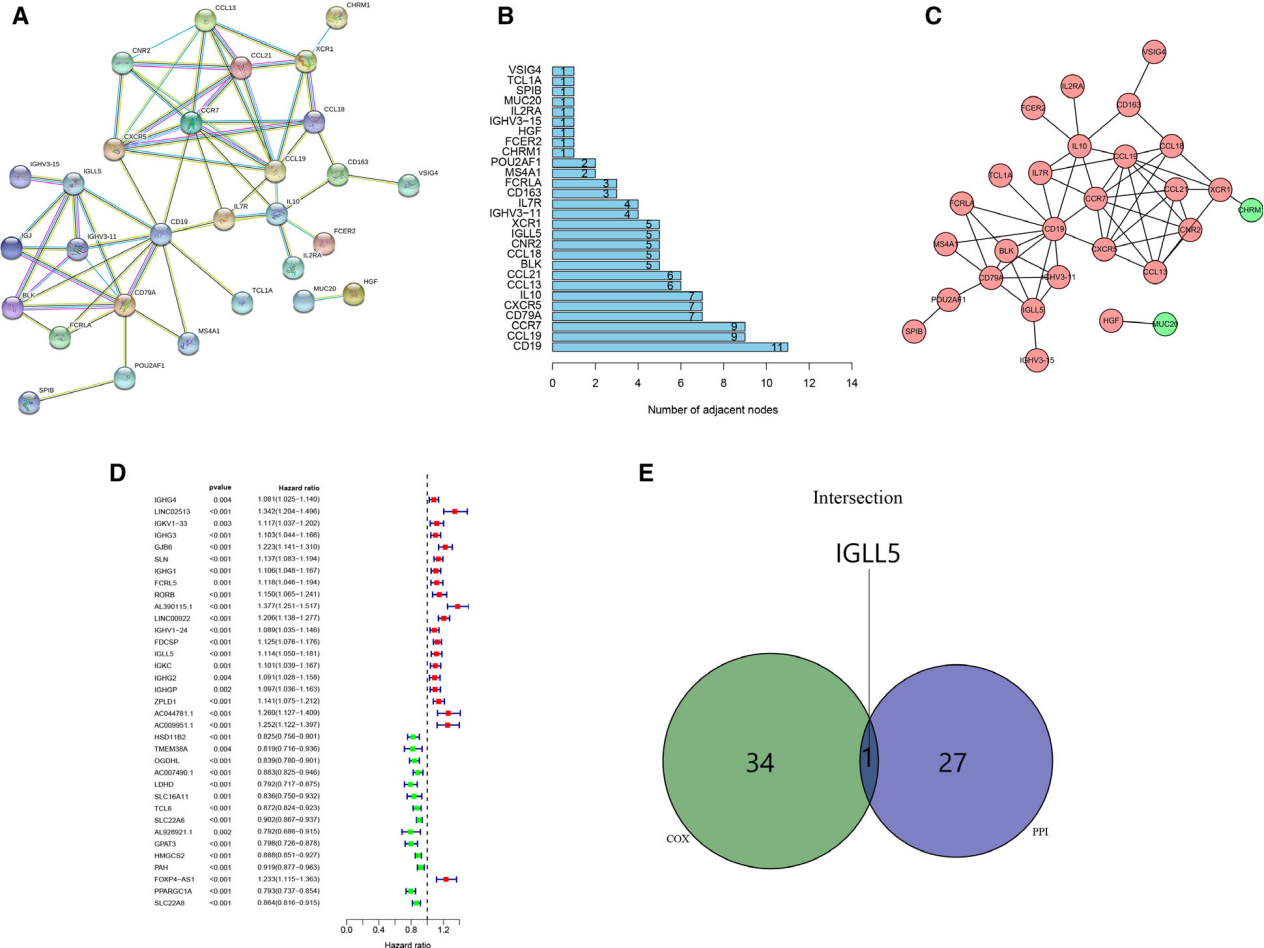
Construction of the PPI network of DEGs and identification of the prognostic hub genes. (A) A PPI network of DEGs with interaction confidence value >0.70. (B) Hub genes ranked by the number of nodes. (C) Network indicated the UR and DR genes using the Cytoscape software. (D) Forest plots listing the prognostic genes with *P* < 0.005. (E) A Venn diagram showing the prognostic hub gene in the PPI network and univariate Cox analysis.

For further exploring the interplay of the DEGs, we constructed a PPI network using the STRING online tool with the interaction confidence value >0.70 as the cutoff criterion. The interactions between the intersecting DEGs are shown in Fig. 5A, and we ranked hub genes on the basis of the number of nodes, as depicted in the bar plots (Fig. 5B). Then we analyzed these 28 hub genes via the Cytoscape software and found 26 UR genes and 2 DR genes (Fig. 5C). Next, we performed the univariate Cox regression analysis of survival to define the prognostic genes among 259 intersecting DEGs and noted that 35 genes were associated with prognosis (Fig. 5D). Finally, an intersection analysis was undertaken between the leading nodes in the PPI network and the prognostic genes, and there was only one overlapping gene, *IGLL5* (Fig. 5E).

### Correlation of *IGLL5* expression with the clinicopathological staging and survival of patients with ccRCC

*IGLL5* located within the immunoglobulin lambda locus encoded one of the *IGLLs*. No correlation was noted between the first exon of this gene and immunoglobulin variable genes; the immunoglobulin lambda joining 1 and the immunoglobulin lambda constant 1 gene segments were taken as the second and third exons, respectively. Diseases associated with *IGLL5* included Schuurs–Hoeijmakers syndrome and retina lymphoma. The gene‐associated GO annotations included antigen binding. The *IGLL5* gene‐associated information was retrieved from https://www.genecards.org.

**Fig. 6:**
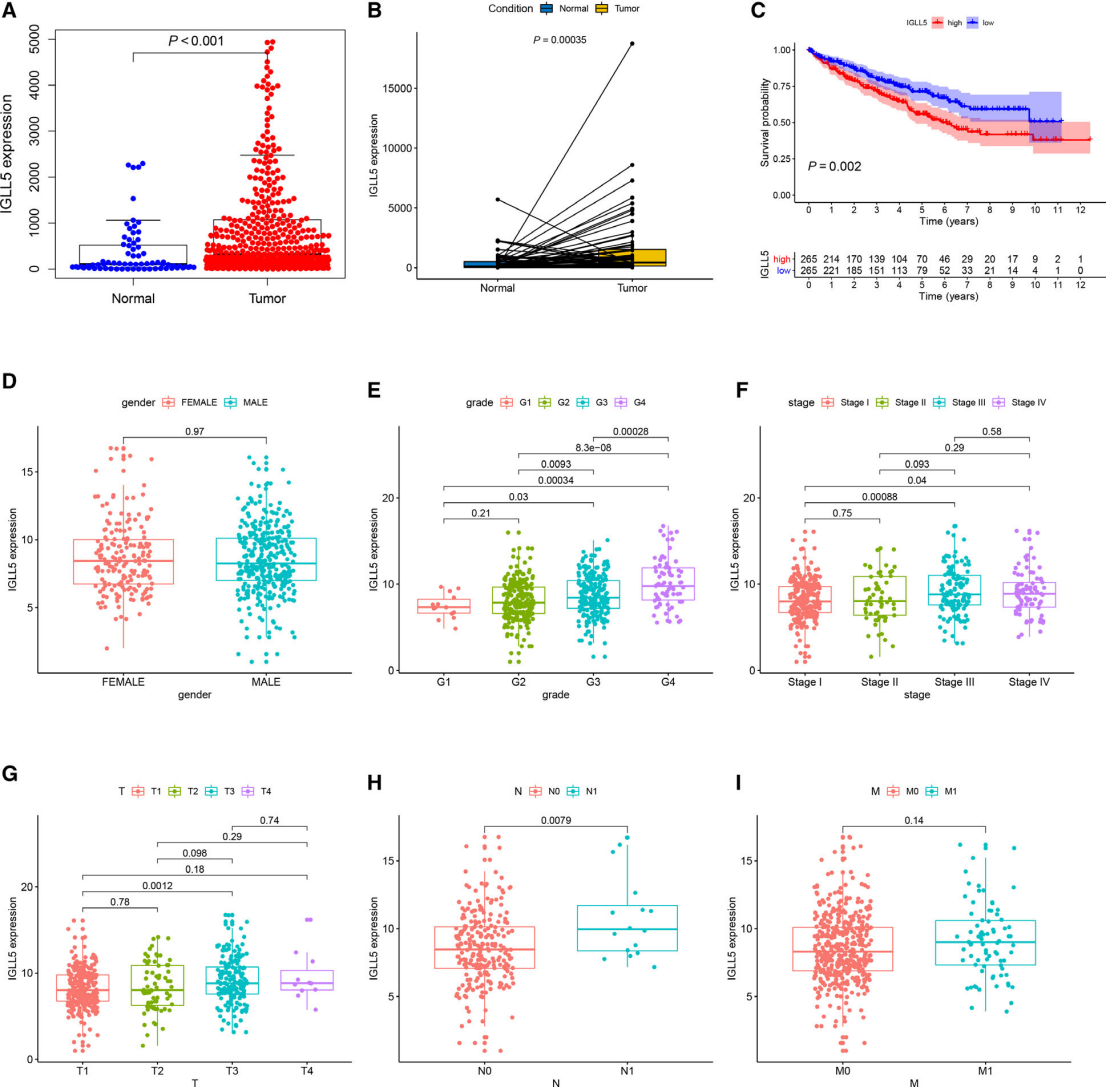
Correlation of IGLL5 expression with the survival and the clinicopathological characteristics of patients with ccRCC. (A) Differential expression analysis of IGLL5 in the normal and ccRCC samples by the Wilcoxon rank‐sum test. (B) Paired differentiation analysis of IGLL5 expression by the Wilcoxon rank‐sum test. (C) Kaplan–Meier survival analysis of H‐IGLL5‐E and L‐IGLL5‐E groups by the log‐rank test. (D–I) The correlation analyses of IGLL5 expression with clinicopathological characteristics. The IGLL5 expression increased with Fuhrman grade (G1–G4), clinical stage (stage I–IV), tumor size (T1–T3) and the number of lymph node metastases (*P* < 0.05).

Differential expression analysis of *IGLL5* between normal samples and ccRCC samples showed that *IGLL5* expression was higher in tumor samples (*P* < 0.001; Fig. 6A,B). Then we grouped all ccRCC samples into high *IGLL5* expression (H‐IGLL5‐E) group and low *IGLL5* expression (L‐IGLL5‐E) group on the basis of median IGLL5 expression. According to survival analysis, ccRCC samples with L‐IGLL5‐E had longer survival than those with H‐IGLL5‐E (*P* < 0.005; Fig. 6C). After comparing the clinicopathological parameters with the expression of *IGLL5* by the Wilcoxon rank‐sum test, we found that the expression of *IGLL5* increased with Fuhrman grade (G1–G4), clinical stage (stage I–IV), tumor size (T1–T3) and the number of lymph node metastases (*P* < 0.05), whereas the differences in sex and distant metastases were not statistically significant (Fig. 6D–I). The results suggested that *IGLL5* had a correlation with the occurrence and development of tumor, and the UR expression of *IGLL5* indicated negative prognosis of ccRCC.

### GSEA enrichment analyses of *IGLL5*

**Fig. 7:**
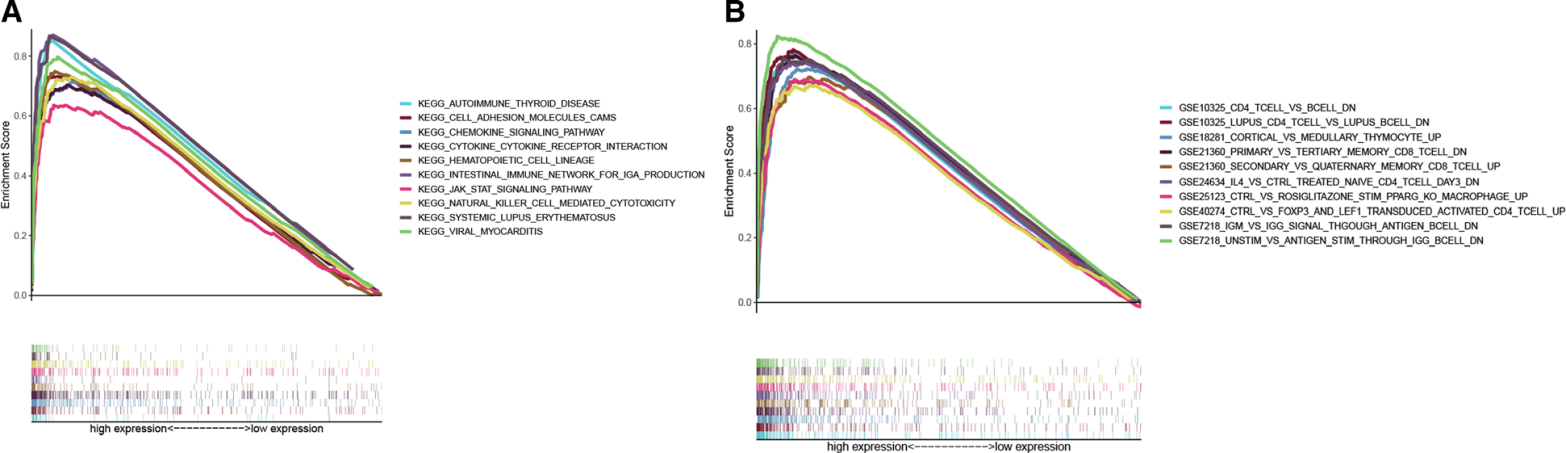
GSEA enrichment analyses for IGLL5. (A) The multi‐GSEA enrichment plot of KEGG collection in H‐IGLL5‐E group (FDR < 0.001). Each particular gene set was represented by one line with unique color, and UR genes were placed on the left of the *x*‐axis, while the DR genes were on the right side. (B) The multi‐GSEA enrichment plot of C7 collection defined in the H‐IGLL5‐E group (FDR < 0.001).

To better comprehend the role of *IGLL5* in ccRCC, we carried out gene set enrichment analyses (GSEA) on H‐IGLL5‐E and L‐IGLL5‐E groups that were divided by the median level of *IGLL5* expression. In C2 collection defined by MSigDB, KEGG gene sets, the genes in the H‐IGLL5‐E group were mostly enriched in autoimmune diseases, cell adhesion molecules, chemokine signaling pathway, cytokine–cytokine receptor interaction, hematopoietic cell lineage, intestinal immune network for immunoglobulin A production, PI3K (Phosphoinositide 3‐kinase) –Akt signaling pathway, natural killing cell‐mediated cytotoxicity, among others (Fig. 7A). In the C7 collection, numerous immune functional gene sets were enriched in the H‐IGLL5‐E group (Fig. 7B). We selected the top 10 gene sets according to FDR (<0.001) and drew the multi‐GSEA enrichment plot. However, few gene sets were enriched in the L‐IGLL5‐E group in both KEGG and C7 collections (there were no gene sets with FDR *q* < 0.5).

### Correlation of *IGLL5* expression with TICs

**Fig. 8:**
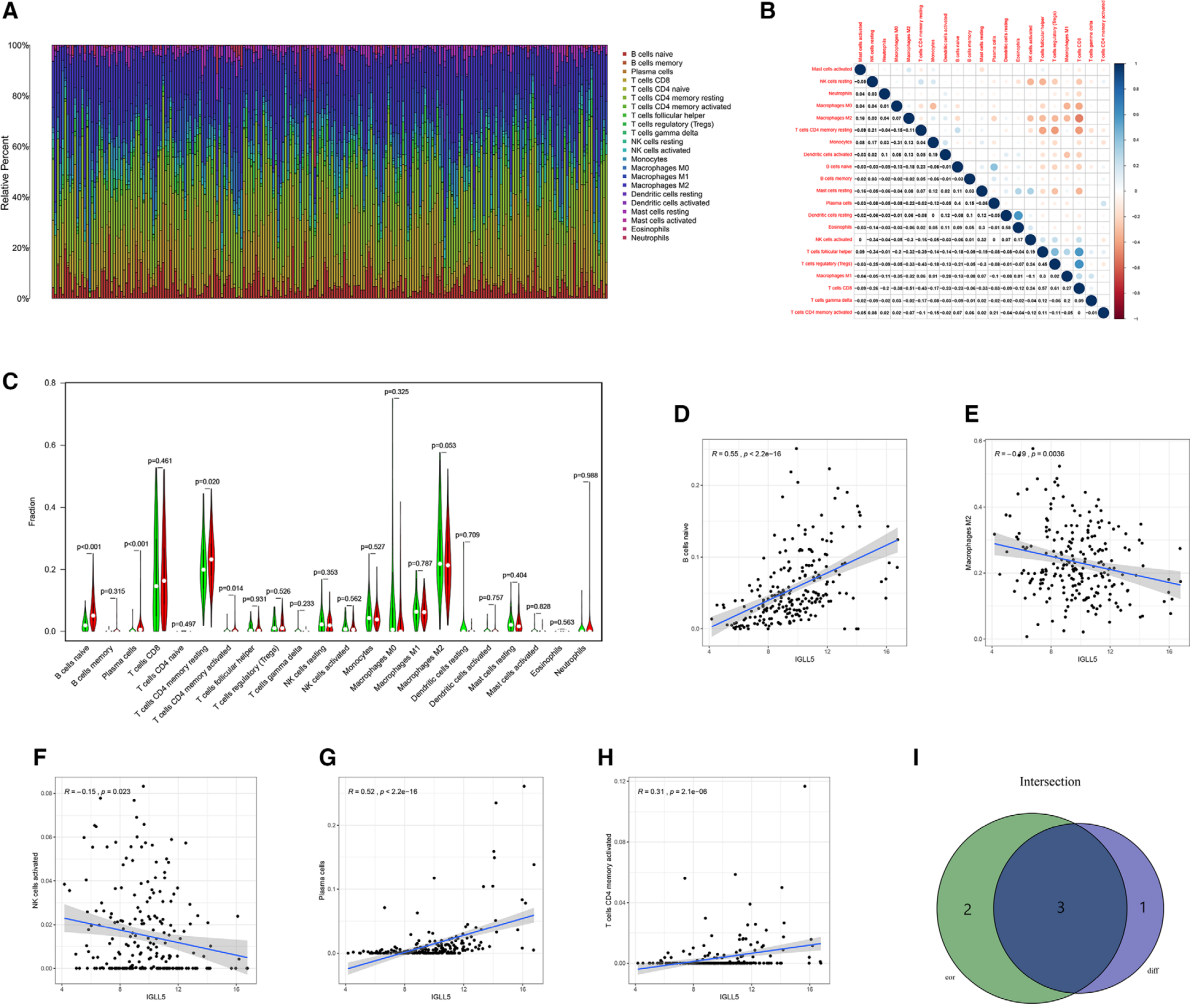
Correlation of IGLL5 expression with TICs. (A) The bar plot shows the proportion of 22 types of TIC in ccRCC samples. (B) The heatmap displays the correlation among 22 types of TIC. (C) The violin plot depicts the fractions of 22 types of immune cell in ccRCC samples between H‐IGLL5‐E and L‐IGLL5‐E groups according to the median expression level of IGLL5, and differences in four types of TIC were statistically significant (*P* < 0.05) by the Wilcoxon rank‐sum test. (D–H) The scatterplots show the correlation among five types of TIC and the IGLL5 expression level with *P* < 0.05 by Spearman's rank correlation test. (I) A Venn diagram illustrates that three types of TIC were correlated with IGLL5 expression.

We implemented further analyses to verify the correlation of *IGLL5* expression with the TME via the CIBERSORT package in r programming language. Figure 8A shows the establishment of 22 types of immune cell profile in ccRCC samples, and the association between these TICs was displayed by heatmap (Fig. 8B). Each intersection represented the correlation between the two immune cells, which was expressed by *P* value and the shade of the circle. Then we conducted the difference analysis to compare the proportions of TICs in the H‐IGLL5‐E group with those in the L‐IGLL5‐E group, and we found that differences in naive B cells, plasma cells, resting memory T cells, and activated CD4 memory T cells were statistically significant (*P* < 0.05; Fig. 8C). Similarly, Spearman's rank correlation test was used, and we found that differences in naive B cells, plasma cells, activated CD4 memory T cells, activated NK cells and M2 macrophages were statistically significant (*P* < 0.05; Fig. 8D–H). The results showed that three types of TIC were positively correlated with *IGLL5* expression (Fig. 8I), and *IGLL5* expression affected the immunological parameters associated with TME.

## Discussion

A number of scholars have universally introduced RCC as the third most frequent kind of urological neoplasm, which causes thousands of deaths every year. However, prognostic and therapeutic biomarkers of RCC have not yet been identified.

In recent years, TME has rapidly attracted the attention of the tumor research community and has been recognized as a key factor in tumorigenesis and development, therapeutic resistance and prognosis [5, 6, 7]. It is widely regarded that RCC has a unique immune microenvironment. Perhaps more than any other solid tumor type, RCC is infifiltrated with immune cells of various phenotype and function [20, 21, 22]. Some studies have shown that TICs in TME are such a promising indicator of prognosis [9, 23]. Since the appearance of tyrosine kinase inhibitors that could antagonize vascular endothelial growth factor receptor to treat metastatic RCC in 2005, the treatment of RCC has entered the era of targeted therapy [24, 25, 26, 27]. However, tyrosine kinase inhibitors have poorer clinical effects on the treatment of RCC with higher level of TICs, and the objective response rate, clinical benefit rate, and OS were worse in patients with RCC with higher TICs [9]. With the advent of immunotherapy, the efficacy of targeted therapy has markedly improved. The combination of vascular endothelial growth factor receptor inhibitors and immune checkpoint inhibitors (ICIs) can help patients to tolerate the treatment well and prolong the survival of patients with RCC [28, 29]. Immunotherapy, especially ICI, has shown promising clinical outcomes in patients with several solid tumors [30]. Several types of immunotherapy are currently thought to work by evoking immune responses in TME [31, 32]. It was reported that compositions of TICs are correlated with effects on the immunotherapeutic interventions [10]. The earlier‐mentioned studies confirmed the effects of TICs on the treatment of RCC.

In this study, we attempted to explore the prognosis of TME‐related genes, and we demonstrated the correlation between the genes and TICs retrieved from TCGA database. We used ESTIMATE to score each ccRCC sample and divided samples into HS and LS groups according to the median score. The results suggested that the score of immune components in TME could better reflect the prognosis of patients with ccRCC. The patients with ccRCC with high immune component had poorer survival and higher histological and clinical stages. Other evidence also suggested that high immune cell infiltration predicted poor prognosis [23]. Next, we identified TME‐related DEGs between the HS and LS groups and screened a total of 259 intersecting genes, consisting of 189 UR and 70 DR genes. The results of GO and KEGG functional enrichment analyses demonstrated that the DEGs were mainly enriched in tumor immune response and ccRCC tumorigenesis. To further explore the interplay of the DEGs, we constructed a PPI network with the interaction confidence value of >0.70 as the cutoff criterion, and we consequently detected 28 hub genes. Afterward, we identified 35 prognosis‐related genes using univariate Cox regression analysis. Finally, *IGLL5* could be detected, which is a gene that overlapped in the hub genes in the PPI network and the prognosis‐related genes.

*IGLL5* located within the immunoglobulin lambda locus encoded one of the IGLLs, which were the immunoglobulin lambda joining 1 and the immunoglobulin lambda constant 1 gene segments. Only a limited number of research has shown that *IGLL5* has a frequent mutation in chronic lymphocytic leukemia [33, 34], lymphoma [35, 36, 37], and multiple myeloma [38, 39]; besides, *IGLL5* has a prognostic value in several diseases, such as multiple myeloma and glioblastoma [39, 40, 41]. Moreover, previous studies have shown that *IGLL5* has a prognostic value in ccRCC, while no in‐depth study has concentrated on TICs.

We used CIBERSORT to calculate the relative proportion of each TIC in a tumor sample. We further conducted differential analysis and correlation analysis to explore the correlation between *IGLL5* and TICs. The results revealed that the expression of *IGLL5* was correlated with three types of TIC ratio: naive B cells, plasma cells and activated CD4 memory T cells. The main role of tumor‐infiltrating B cells has been found to be controversial, and studies suggested that they play a tumor‐promoting role by suppression of the antitumor response [42, 43]. Inhibition of *IGLL5* aiming to reduce the infiltration of B cells in tumors may provide a new target for combined immunotherapy.

In summary, *IGLL5*, a TME‐related hub gene, was extracted from functional enrichment analysis of TCGA database based on ESTIMATE algorithm. After survival analysis and evaluation of prognostic values, *IGLL5* may become potential a prognostic biomarker of ccRCC. Combined immunotherapy based on *IGLL5* inhibitor and ICIs provides a new idea and needs further study.

## Materials and methods

### TCGA data

Transcriptome RNA sequencing data and the corresponding clinical data of 611 ccRCC cases (normal samples, 72 cases; tumor samples, 539 cases) were downloaded from TCGA database (https://portal.gdc.cancer.gov/), and the samples with unknown information were removed.

### ESTIMATE algorithm

r programming language version 3.6.2 (https://www.r‐project.org/) was used for estimating ratio of immune‐stromal component in TME for each sample by ESTIMATE algorithm. ESTIMATE algorithm involved three types of score: ESTIMATEScore, StromalScore and ImmuneScore.

### Survival analysis and difference analysis of scores with clinicopathological staging

r language loaded with package survival and survminer was used for the survival analysis, and the survival curve was plotted by Kaplan–Meier method. We considered *P* < 0.05 statistically significant.

The Wilcoxon rank‐sum test was used to compare differences between scores and clinicopathological characteristics.

### Identification of DEGs

In addition, division of 539 tumor samples into HS and LS groups was undertaken on the basis of the median score. DEGs with FDR <0.05 and fold change (FC) >1 after transformation into log_2_ (HS group/LS group) were considered significant.

### Heatmaps

We utilized r programming language, especially pheatmap package, to plot heatmaps of DEGs.

### GO and KEGG enrichment analysis

We used r programming language to conduct GO and KEGG enrichment analyses of 259 DEGs with the aid of the packages clusterProfifiler, ggplot2 and enrichplot. Terms with *P* < 0.05 were considered significantly enriched.

### PPI network construction

To further explore the interplay of the DEGs, we attempted to construct a PPI network with the aid of the STRING online tool, and a cutoff criterion was taken into account (minimum required interaction score > 0.70). Then we reconstructed the network to label UR and DR genes with cytoscape of version 3.6.1 (https://cytoscape.org).

### Cox regression analysis

The univariate Cox regression analysis was performed on survival to define the prognostic genes among 259 intersecting DEGs, and we found that 35 genes were associated with prognosis, listing in the forest plots with *P* < 0.005.

### GSEA

We attempted to carry out GSEA enrichment analyses of C2‐KEGG and C7 gene sets with the aid of gsea 4.0.3 software (http://software.broadinstitute.org/gsea/index.jsp).

### TICs profile

In tumor samples, for estimation of the TIC abundance profile, the CIBERSORT method was used.

## Conflict of interest

The authors declare no conflict of interest.

## Author contributions

CZ and Z‐NX conceived and supervised the study; Z‐NX and X‐YW designed and performed experiments; Z‐NX and L‐CC analyzed data; and Z‐NX and W‐GJ wrote the manuscript.

## Data Availability

Data will be available from the corresponding author upon reasonable request.
